# Continuous Time-Domain Cerebrovascular Reactivity Metrics and Discriminate Capacity for the Upper and Lower Limits of Autoregulation: A Scoping Review of the Animal Literature

**DOI:** 10.1089/neur.2021.0043

**Published:** 2021-12-20

**Authors:** Amanjyot Singh Sainbhi, Logan Froese, Alwyn Gomez, Carleen Batson, Kevin Y. Stein, Arsalan Alizadeh, Frederick A. Zeiler

**Affiliations:** ^1^ Biomedical Engineering, Faculty of Engineering, University of Manitoba, Winnipeg, Manitoba, Canada.; ^2^ Section of Neurosurgery, Department of Surgery, Rady Faculty of Health Sciences, University of Manitoba, Winnipeg, Manitoba, Canada.; ^3^ Department of Human Anatomy and Cell Science, Rady Faculty of Health Sciences, University of Manitoba, Winnipeg, Manitoba, Canada.; ^4^ Centre on Aging, University of Manitoba, Winnipeg, Manitoba, Canada.; ^5^ Division of Anaesthesia, Department of Medicine, Addenbrooke's Hospital, University of Cambridge, Cambridge, United Kingdom.

**Keywords:** cerebrovascular autoregulation, cerebrovascular reactivity metrics, Lassen autoregulatory curve, lower limit of autoregulation, time-domain metrics

## Abstract

Over a wide range of systemic arterial pressures, cerebral blood flow (CBF) is regulated fairly constantly by the cerebral vessels in a process termed cerebral autoregulation (CA), which is depicted by the Lassen autoregulatory curve. After traumatic brain injury (TBI), CA can get impaired and these impairments manifest in changes of the Lassen autoregulatory curve. Continuous surrogate metrics of pressure-based CA, termed cerebrovascular reactivity (CVR) metrics, evaluate the relationship between slow vasogenic fluctuations in a driving pressure for cerebral blood flow, and the most commonly studied and utilized measures are based in the time domain and have been increasingly applied in bedside TBI care and have sparked the investigation of individualized cerebral perfusion pressure targets. However, not all CVR metrics have been validated as true measures of autoregulation in the pre-clinical setting. We reviewed all available pre-clinical animal literature that assessed the association between continuous time-domain metrics of CVR and some aspect of the Lassen autoregulatory curve. All 15 articles found associated the evaluated continuous metrics to the lower limit of autoregulation curve whereas none looked at the upper limit. Most of the evaluated metrics showed the ability to discriminate the lower limit of autoregulation with various methods of perturbation. Further work is required to evaluate the utility of such surrogate measures against the upper limit of autoregulation, while also providing validation to the existing literature supporting specific indices and their ability to discriminate the lower limit.

## Introduction

The innate ability of the cerebral vessels to maintain cerebral blood flow (CBF), relatively constantly, over a wide range of systemic arterial pressures, is termed cerebral autoregulation (CA).^1,2^ The Lassen autoregulatory curve depicts CA where the CBF is relatively constant between the lower and upper limits of autoregulation (LLA and ULA). The Lassen autoregulatory curve is plotted with cerebral perfusion pressure (CPP) or mean arterial pressure (MAP) along the x-axis, and CBF along the y-axis, where CBF has classically been invasively measured.^3^

Impairment in CA has been documented in various neuropathological states, including post-stroke^4–7^ and after traumatic brain injury (TBI),^8–14^ across the spectrum of injury severity. Such impairment manifests in changes in the position of the LLA or ULA on the Lassen autoregulatory curve or, in the worst instance, absence of the autoregulatory curve altogether.^7,15^ These alterations in the autoregulatory curve expose the brain to pressure-passive flow states, where low MAP or CPP leads to hypoperfusion, whereas high MAP or CPP leads to hyperperfusion. Recent literature suggests that the exposure burden of impaired CA is a significant driver of poor long-term outcomes in various neurological conditions.^10–12,16–18^ Subsequently, to avoid subjecting the brain to hypo- or hyperperfusion conditions, it is imperative that we have the capacity to continuously and accurately monitor CA at the bedside.

Direct measurement of CA is not possible at the bedside, given that continuous and accurate measures of CBF are not readily available to the treating clinician. As such, we must rely on surrogate metrics of CA, termed cerebrovascular reactivity (CVR) metrics, involving changes in gas levels, pressure, or flow velocity to drive alterations in vascular caliber.^8,15,19^ Continuous indices that have not been fully validated as measures of the Lassen autoregulatory curve have been given the term CVR metrics. The clinical realm has made a concerted effort to refer to these indices as CVR metrics to avoid misrepresenting them as a completely validated measure of autoregulation. We will maintain such consistent nomenclature throughout this article in keeping with this designation within the field. Such continuous CVR metrics evaluate the relationship between slow vasogenic fluctuations in a driving pressure for CBF (i.e., MAP or CPP) and a surrogate for pulsatile cerebral blood volume (CBV) or CBF. The table in Supplementary Appendix SB provides a list of continuous CVR metrics derived from various cerebral monitoring techniques, which are of interest given that they can be adopted in the bedside clinical care of TBI patients. As such, CVR indices of interest are only those that have continuously updating data streams.

The most readily studied and utilized measures are based in the time domain and are derived as moving Pearson's correlation coefficients between raw physiological signals, with negative values denoting “intact” autoregulation and positive values denoting “impaired” autoregulation. Cerebral monitoring devices utilized to obtain surrogate measures for pulsatile CBV/CBF derived from raw continuous physiological signals include both invasive and non-invasive modalities, such as: intracranial pressure (ICP), near-infrared spectroscopy (NIRS), transcranial Doppler (TCD), brain tissue oxygen (PbtO_2_), thermal diffusion flowmetry (TDF) CBF, and laser Doppler flowmetry (LDF) CBF.^19,20^ With these devices, various CVR indices can be derived at the bedside in humans, with most literature to date focused on TBI patient cohorts. We mainly focused on time-domain metrics given that these measures have seen widespread adoption by clinicians at the bedside because they give greater simplicity in continuous derivation and interpretation over frequency-domain measures. However, not all indices have been explored in pre-clinical models as measures of the Lassen autoregulatory curve, and, as such, we conducted this study.

However, recent literature in moderate/severe TBI suggests that not all such continuously derived CVR metrics are the same, with varying levels of covariance between them.^21–23^ Further, literature validating these metrics against the Lassen autoregulatory curve in pre-clinical models has been scarce and scattered across various subspecialty journals.^19,20,24,25^ This ambiguity has left confusion among clinicians as to which indices are truly validated measures of autoregulation, despite support for their use from international consensus groups.^14,26–28^ Thus, it is imperative that we understand which measures truly evaluate aspects of the Lassen autoregulatory curve and which do not. Such knowledge will improve confidence in their use to manage TBI patients and highlight areas for future work. As such, the goal of this study was to perform a systematically conducted scoping review of the pre-clinical animal literature, assessing for any documented association between continuous time-domain metrics of CVR and some aspect of the Lassen autoregulatory curve.

## Methods

A systematically conducted scoping review of the available literature was conducted using the methodology outlined in the Cochrane Handbook for Systematic Reviews.^29^ The data were reported in line with the Preferred Reporting Items for Systematic Reviews and Meta-Analysis (PRISMA).^30^ Supplementary Appendix SA of the Supplementary Materials provides the PRISMA checklist. The search strategy and methodology are similar to other scoping reviews published by our group.^31–33^ Review questions and search strategy were decided upon by the supervisor (F.A.Z.) and the primary author (A.S.S.).

### Search questions, population, and inclusion and exclusion criteria

The question posed for this scoping systematic review was: What pre-clinical animal literature exists to validate continuous measures of CVR as true measures of the Lassen autoregulatory curve? All studies, either prospective or retrospective, of any size were included.

The primary outcome measure was the association between continuous CVR measures and the Lassen autoregulatory curve.^3^ Continuous CVR measures were defined as those which are moving Pearson's correlation coefficients between slow-wave fluctuations in a driving pressure for CBF (i.e., either MAP or CPP) and a surrogate for pulsatile CBV/CBF, as defined in the existing literature body.^19,20^ The following cerebral monitoring techniques were considered eligible for the derivation of continuous CVR metrics: ICP, NIRS, TCD, PbtO_2_, TDF CBF, or LDF CBF. Supplementary Appendix SB provides a table of the CVR metrics derived from these devices which were of interest in this review.

All studies, whether prospective or retrospective, of all sizes, including any animal model type that evaluated time-domain continuous metrics of pressure-based CVR and documented an association between the continuous metric and some aspect of the Lassen autoregulatory curve were eligible for inclusion in this review. Exclusion criteria were the following: being non-English-language studies, human studies, non-continuous CVR assessments, and non-pressure-based CVR measures (i.e., chemoreactivity or CO_2_ reactivity testing).

### Search strategy

MEDLINE, BIOSIS, EMBASE, Global Health, SCOPUS, and the Cochrane Library from inception to the end of January 2021 were searched using individualized search strategies for each database. The search strategies for all the databases can be seen in Supplementary Appendix SC of the Supplementary Materials. Finally, the reference lists of reviewed articles on CVR were examined to ensure that no references were left out.

### Study selection

Using two reviewers (A.S.S. and L.F.), a two-step review of all articles returned by our search strategies was performed. First, the reviewers independently screened all titles and abstracts of the returned articles to decide whether they met the inclusion criteria. Second, the full text of the chosen articles was assessed to confirm whether they met the inclusion criteria, and that the primary outcome of CVR was documented. Finally, any discrepancies between the two reviewers were resolved by a third party (F.A.Z.).

### Data collection

Data fields included the following: animal model details, the study's goal, aspects of Lassen autoregulatory curve assessed, primary/secondary outcomes, limitations, CVR indices measured, method of perturbation, and conclusions regarding continuous indices.

### Bias assessment

Given that the goal of this review was to provide a comprehensive scoping overview of the available literature, a formal bias assessment was not conducted.

### Statistical analysis

A meta-analysis was not performed in this study because of the heterogeneity of study designs and data.

## Results

### Search results and study characteristics

**FIG. 1.**
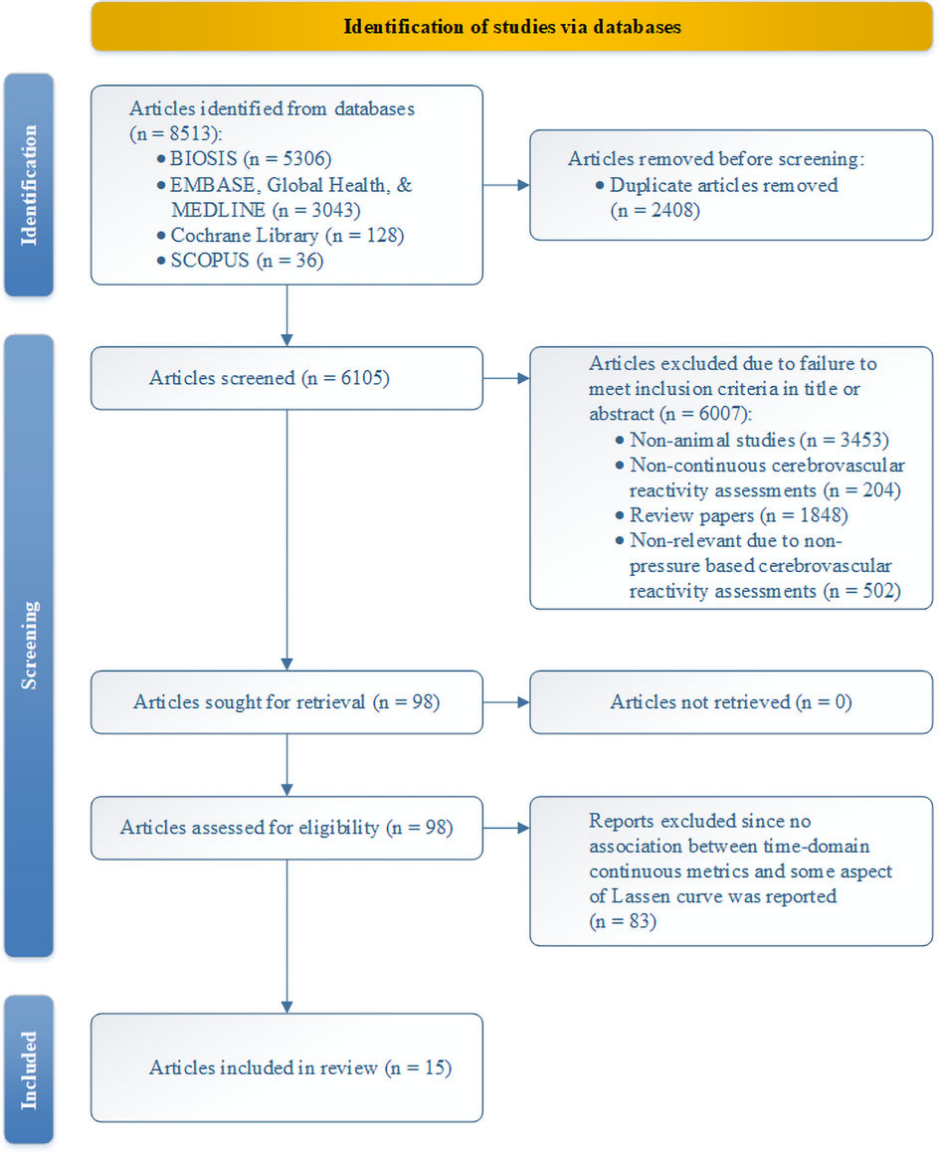
PRISMA flow diagram. PRISMA, Preferred Reporting Items for Systematic Reviews and Meta-Analysis.

Results of the search strategy across all databases and other sources are summarized in Figure 1. There were a total of 8513 articles identified from the databases searched. A total of 2408 articles were removed because of duplicated references, leaving 6105 articles to review. By applying the inclusion/exclusion criteria to the title and abstract of these articles, we identified 98 articles that fit these criteria. No articles were added from reference sections of pertinent review articles, which left 98 articles to review. On applying the inclusion/exclusion criteria to the full-text documents, only 15 were found eligible for inclusion in the systematic review, all from the database search. Articles were excluded because they either did not report details around the association of time-domain continuous metrics and some aspect of the Lassen autoregulatory curve, were review articles, were non-animal literature, or were non-relevant given that they reported on non-pressure-based CVR measures.

Supplementary Appendix SD gives a general overview table of all 15 animal studies with primary and secondary outcomes of the study and its limitations.^19,20,25,34–45^ Tables 1, 2, and 3 describe three categories of perturbation methods: arterial hypotension/hypertension,^19,25,34–41^ ICP perturbation,^20,35,43,45^ and other methods of inducing perturbation (such as cardiac arrest, hypothermia, etc.),^25,37,38,40–42,44,45^ respectively. Articles that used mixed methods will be found in more than one of Tables 1, 2, and 3 along with the study's conclusions regarding continuous indices. To measure CBF, 12 studies used LDF,^19,20,25,34,35,38–44^ two studies used laser Doppler index (LDx),^36,37^ and one study used a diffusion coefficient measured with a custom-built diffusion coefficient spectroscopy.^45^ All eligible studies reported the association between time-domain continuous metrics and the LLA aspect of the Lassen's autoregulatory curve. Only one study fully reconstructed the Lassen autoregulatory curve with both the LLA and ULA, but only related the continuous metrics to the curve's plateau (the region between LLA and ULA).^45^

**Table 1.** Performance of Continuous Cerebrovascular Reactivity Indices: Arterial Hypotension/Arterial Hypertension Studies Only

References	Animal model details	CVR indices measured	Method of inducing hypo-/hypertension	Aspects of the Lassen autoregulatory curve measured	Conclusions regarding continuous indices
Brady et al. (2007)^34^	Piglets 3–8 days old and weighing 2.2–3.9 kg. 6 piglets were made progressively hypotensive (low blood pressure) over 4–5 h.	COx LDx	Hypotension was induced by gradual inflation of a 5-French esophageal balloon catheter, interrupting the venous return to the heart over 4–5 h.	LLA: • A scatterplot of LDF vs. CPP was made with all data for each piglet. • CPP at the intersection of two regression lines with the lowest combined residual squared error was defined as the autoregulatory breakpoint.	Both indices accurately described the autoregulatory breakpoint well, while LDx performed better than COx. When evaluated on a minute-to-minute basis, the agreement between the indices was limited (Pearson's *r* = 0.36), but it greatly improved by averaging values (Pearson's *r* = 0.67). The Bland-Altman method showed −0.06 bias for all values measured and 0.03 for averaged values.
Brady et al. (2008)^35^	Piglets were 5–10 days old and weighing 2.2–3.9 kg. 8 piglets were in the naïve ICP group (ICP not altered). 6 piglets were in the elevated ICP group (ICP altered to ≈20 mm Hg). Arterial hypotension: low blood pressure induced by a balloon catheter	PRx COx LDx	Arterial hypotension was induced by a balloon catheter in the inferior vena cava.	LLA: • A scatterplot of 1-min-averaged LDF vs. CPP was made for each piglet. • CPP at the intersection of two regression lines with the lowest combined residual squared error was defined as the autoregulatory breakpoint.	Similar values obtained above LLA using the three indices, under both naïve and elevated ICP conditions, that suggests, in the uninjured brain, that the autoregulatory mechanism is not improved by driving CPP in excess of LLA. All three indices are accurate in the detection of loss of autoregulation attributable to hypotension. COx and LDx assessed progressive pressure passivity caused when CPP was below LLA. PRx-derived autoregulation curves comparative to COx- and LDx-derived autoregulation curves have a blunted response at CPP around LLA. At either naïve or elevated ICP, PRx was very specific for loss of autoregulation. PRx accuracy was not dependent on intracranial compliance.
Brady et al. (2010)^36^	Waveform recordings of 25 piglets 5–7 days old • Identified and included from past studies with naïve ICP along with intact recordings of ICP, MAP, and red cell flux in parietal cortex using laser Doppler and rSO_2_	COx COx-a	Arterial hypotension was induced by gradually decreasing MAP by infusion into the balloon catheter in the inferior vena cava over 158 ± 50 min.	LLA: • Continuous red cell flux, recorded from laser Doppler, was plotted as a function of MAP. • These plots were dischotomized to give two best-fit lines having the lowest combined residual error squared, and MAP at their intersection was defined as the LLA. • MAP at LLA was chosen as the gold standard instead of CPP at LLA, given that this study sought to evaluate whether COx can detect MAP above and below LLA without an ICP monitor.	COx-a accuracy is highly comparable to COx (*r* = 0.96, *p* < 0.0001). Data indicate that MAP-derived COx results in a higher threshold COx value of 0.5 (89% sensitive, 81% specific) for discriminating LLA compared to CPP-derived COx, which has a threshold value of 0.42 (89% sensitive, 77% specific) in the same animals.
Brady et al. (2012)^37^	10 neonatal swine	PRx iPRx Δϕ_AI_	Hypotension occurred with the gradual hemorrhage by syringe-pump withdrawal at the rate of 12% calculated blood vol/h.	LLA: • Continuous cortical laser Doppler flux was used to delineate the LLA. • Flux measurements were plotted across CPP to determine LLA by piece-wise linear regression at the intersection of two best-fit lines with the lowest residual error squared.	PEEP modulation significantly improved the precision of PRx monitoring. At normal MAP: • Before PEEP oscillations, the resultant PRx was −0.06. • With PEEP oscillations: ○ iPRx at normotension was −0.39, and at hypotension above LLA was −0.42 while below LLA was 0.32 (*p* = 0.0004). ○ Δϕ_AI_ at normotension was 150 degrees, and at hypotension above LLA was 161 degrees while below LLA was −31 degrees (*p* < 0.0001). For CPP below LLA: • iPRx threshold value of −0.04 was both 95% sensitive and 95% specific. • Δϕ_AI_ had a phase-angle difference of <115 degrees and was 95% sensitive and <103 degrees was 95% specific. Δϕ_AI_ was significantly lower, causing significantly lower iPRx, during hypotension below LLA compared to the normal pre-load state and mild hypotension where iPRx and Δϕ_AI_ were not different.
Larson et al. (2013)^38^	Neonatal male piglets were 3–5 days old and weighing 1.0–2.5 kg. 48 piglets were randomized into four groups, each containing 12 piglets: • HA injury with hypothermia • HA injury with hypothermia and rewarming • Sham surgery with hypothermia • Sham surgery with hypothermia and rewarming Hypotension was induced in half of the piglets (6) in each of the four groups, and hypertension was induced in the other half of the piglets (6).	COx HVx	Hypotension induced by a 5-French balloon catheter in the inferior vena cava by a femoral vein Cephalic hypertension induced by a balloon catheter placed in the descending aorta by a femoral artery	LLA: • LDF was plotted as a function of CPP. • CPP at the intersection of two regression lines with the lowest combined residual squared error was defined as the LLA.	COx (*p* > 0.10) and HVx (*p* > 0.05) accurately detected differentiated CPP below and above the LLA. Both HVx and COx increased as CPP decreased. During hypertension, cerebral autoregulation remained functional after HA injury after hypothermia or hypothermia and rewarming.
Lee et al. (2009)^39^	8 piglets 5–10 days old and weighing 2.34 ± 0.47 kg Hypotension was induced in these piglets.	HVx PRx	Slow, controlled systemic hypotension was induced by slowly inflating the 5-French esophageal balloon catheter in the inferior vena cava.	LLA: • A scatterplot of 1-min-averaged LDF vs. CPP was generated for each piglet. • CPP at the intersection of two regression lines with the lowest combined residual squared error was defined as the LLA.	Generally, both HVx and PRx were higher below the LLA, indicating pressure passivity, and lower above the LLA, indicating pressure reactivity (*r* = 0.73). HVx is an excellent alternative to PRx.
Lee et al. (2011)^40^	64 neonatal male swine were 3–5 days old and weighing 1.0–2.5 kg. Piglets were divided into eight groups: • Hypotensive cohorts ○ Post-arrest normothermia ○ Post-arrest hypothermia ○ Sham normothermia ○ Sham hypothermia • Hypertensive cohorts ○ Post-arrest normothermia ○ Post-arrest hypothermia ○ Sham normothermia ○ Sham hypothermia	COx HVx	Arterial hypotension was induced by decreasing MAP over 3 h by inflating a 5-French balloon catheter in the inferior vena cava. Hypertension was induced by increasing MAP over 3 h by inflating an aortic balloon catheter and infusing phenylephrine and dopamine.	LLA: • LDF plotted as a function of CPP • LLA is defined as the CPP at the intersection of two linear regression lines resulting in the lowest combined error squared.	HVx and COx were good predictors of whether CPP was above or below the LLA, but the accuracy was slightly less in post-arrest animals. In hypotension cohorts, sham animals with CPP below LLA were detected by COx 0.41 and HVx 0.22 whereas arrested animals with CPP below LLA were detected by COx 0.42 and HVx 0.21. There was a 50% probability that CPP exceeded LLA at the HVx and COx values given above. In hypertension cohorts, as CPP increased, HVx and COx values did not increase above zero and were consistent with preserved autoregulation during post-resuscitation normothermia and hypothermia.
Lee et al. (2012)^41^	Neonatal male piglets were 3–5 days old and weighing 1.0–2.5 kg. To evaluate LLA, 24 piglets were divided into three groups (8 per group): • 1 day of recovery after arrest • 2 days of recovery after arrest • 2 days of recovery after the sham procedure To evaluate autoregulatory response to hypertension, a separate cohort of 10 piglets were divided into two groups (5 per group): • 2 days of recovery after arrest • 2 days of recovery after the sham procedure For neurobehavioral testing and histology, a separate cohort of 11 piglets were put into two groups: • Underwent arrest (6 piglets) • Sham surgeries without cranial instrumentation or blood pressure manipulation (5 piglets)	COx HVx	Hypotension was induced by inflation of a 5-French balloon catheter in the inferior vena cava. Hypertension was induced over 30 min by inflation of an aortic balloon catheter in the descending aorta and infusing phenylephrine and dopamine.	LLA: • LDF was plotted as a function of CPP. • LLA is defined as the CPP at the intersection of two regression lines resulting in the lowest combined error squared.	COx and HVx were good predictors of whether CPP was above or below the LLA. During hypotension, COx and HVx increased as CPP was decreased, with the slope of this relationship being less than zero. • For piglets in the group of 1 day of recovery after arrest, the relationship for COx was biphasic, with the slope below the LLA significantly more negative than the slope above the LLA (*p* = 0.014). During hypertension, the autoregulatory function measured by sROR was similar between piglets that recovered for 2 days after sham surgery and piglets that recovered for 2 days after resuscitation from HA cardiac arrest.
Liu et al. (2020)^25^	Data from 68 neonatal piglets were analyzed from three previous studies where 35 piglets were resuscitated from cardiac arrest and 33 were sham piglets.	PRx wPRx COx wCOx HVx wHVx	Reanalyzed data from three published piglet studies where hypotension was induced by inflation of a 5-French balloon catheter in the inferior vena cava	LLA: • Each piglet's MAP at LLA was identified by ICM+ software, which uses piece-wise regression to dichotomize the data and fit two linear regression lines with the lowest combined error squared. • Each piglet's laser Doppler flow-derived LLA was considered to be the gold standard to test whether autoregulation indices identified MAP above or below the LLA.	All indices discriminated MAP above and below the LLA (*p* < 0.001). Distinguishing MAP above from below LLA did not differ between COx and wCOx or between PRx and wPRx whereas HVx had a significantly higher discriminatory value than wHVx in detecting dysfunctional autoregulation. Wavelet indices highly correlated with their respective correlation indices (*r* = 0.78, *p* < 0.001 for PRx and wPRx; *r* = 0.66, *p* < 0.001 for COx and wCOx; *r* = 0.78, *p* = 0.002 for HVx and wHVx). All the correlation indices became positive and approached +1 as blood pressure deviated farther below the LLA during induced hypotension. The wavelet indices increased as MAP decreased below LLA, but not as much as that observed with the correlation indices. Mean values for all the indices were each significantly lower when MAP was above LLA than below in paired comparisons (*p* < 0.001 for each index). All wavelet indices had lower variability than their corresponding correlation index. The wavelet methodology uses a coherence threshold to eliminate poorly coherent signals to decrease signal noise.
Zeiler et al. (2018b)^19^	Archived data of 22 neonatal piglets were analyzed from three separate experiments: • Control animals from a study on LLA that had 8 piglets which were 5–10 days old and weighed 2.2–3.9 kg • Sham controls for a model of cardiac arrest that had 7 piglets which were 3–5 days old and weighed 1.0–2.5 kg • Sham normothermic controls for a model of cardiac arrest with hypotension therapy that had 7 piglets which were 3–5 days old and weighed 1.0–2.5 kg	PRx PAx RAC	Arterial hypotension was induced by inflation of a 5-French esophageal balloon catheter in the inferior vena cava. Hypotension was induced to near-zero blood pressure over 2–4 h from baseline.	LLA: • Plots of LDF-CBF vs. CPP were constructed using ICM+ software. • Breakpoint representing the LLA for each animal was identified at the intersection of two linear segments with the minimized sum residual squared error with the automated piece-wise linear regression conducted with R statistical software. • Mean LLA was determined by averaging all 22 LLA values obtained for the cohort of 22 piglets.	All three indices appeared to respect the LLA within this model of arterial hypotension, with PRx being superior. PRx (*p* < 0.0001) correlated with LLA in the model of hypotension. Bland-Altman analysis showed poor agreement with LLA. Preliminary evidence shows that both PAx and RAC validate against LLA within a model of hypotension, but strong conclusions cannot be made because of the small number of animals in the current study. The simplistic way of looking at the continuous indices of positive is “bad” and negative is “good” should be avoided given that it was observed that PAx and RAC remained negative even below the LLA until extremely low values of CPP. Clearly, each index is different and requires a detailed evaluation. Caution should be taken when interpreting that CPP at some of the clinically defined index thresholds appears to be related to LLA within this piglet model.

CBF, cerebral blood flow; COx, cerebral-oximetry index; COx-a, COx obtained with MAP; CPP, cerebral perfusion pressure; HA, hypoxic-asphyxic; HVx, hemoglobin volume index; ICM+, Intensive Care Monitoring software (Cambridge Enterprise Ltd, Cambridge, UK); ICP, intracranial pressure; iPRx, induced PRx; LDF, laser Doppler flow; LDF-CBF, LDF-based CBF; LDx, laser Doppler index; LLA, lower limit of autoregulation; MAP, mean arterial pressure; PAx, pulse amplitude index; PRx, pressure-reactivity index; RAC, correlation between pulse amplitude of ICP and CPP; rSO_2_, regional cerebral oximetry; sROR, static rate of autoregulation; wCOx, wavelet COx; wHVx, wavelet HVx; wPRx, wavelet PRx; Δϕ_AI_, MAP-ICP phase shift.

**Table 2.** Performance of Continuous Cerebrovascular Reactivity Indices: ICP Perturbation Studies Only

References	Animal model details	CVR indices measured	Method of inducing ICP change	Aspects of the Lassen autoregulatory curve measured	Conclusions regarding continuous indices
Brady et al. (2008)^35^	Piglets were 5–10 days old and weighing 2.2–3.9 kg. 8 piglets were in the naïve ICP group (ICP not altered). 6 piglets were in the elevated ICP group (ICP altered to ≈20 mm Hg). Arterial hypotension: low blood pressure induced by a balloon catheter	PRx COx LDx	In the elevated ICP group, steady-state ICP of ≈20 mm Hg was maintained by continuous infusion of artificial CSF.	LLA: • A scatterplot of 1-min-averaged LDF vs. CPP was made for each piglet. • CPP at the intersection of two regression lines with the lowest combined residual squared error was defined as the autoregulatory breakpoint.	Similar values obtained above LLA using the three indices, under both naïve and elevated ICP conditions, suggests that the autoregulatory mechanism is not improved by driving CPP in excess of LLA. All three indices are accurate in the detection of loss of autoregulation attributable to hypotension. COx and LDx assessed progressive pressure passivity attributable to CPP below LLA. PRx-derived autoregulation curves comparative to COx- and LDx-derived autoregulation curves had a blunted response at CPP around LLA. At either naïve or elevated ICP, PRx was very specific for loss of autoregulation. PRx accuracy was not dependent on intracranial compliance.
Nusbaum et al. (2014)^43^	Juvenile domestic pigs were divided into two groups: • Naïve ICP group containing 15 piglets with a mean weight of 63.1 kg. • High ICP group containing 20 piglets with a mean weight of 54.2 kg.	• PRx: based on slow-wave changes in measured ICP taken from an invasive ventricular drain • HVx: NIRS-based index	After completion of baseline measurements in the high ICP group, lasting 1 h, a 6F custom latex balloon catheter was gradually inflated in the superior vena cava over 2 h to achieve the elevated ICP of >20 mm Hg. Naïve ICP piglets were maintained at baseline pressure levels for 3 h.	LLA: • A scatterplot of 60-sec-averaged values of LDF vs. CPP was made using the ICM+ software for each piglet. • CPP at the intersection of two regression lines with the lowest combined residual squared error was defined as the autoregulatory breakpoint for each animal.	Both PRx and HVx can differentiate the autoregulatory state of the brain above or below the LLA. The NIRS-based HVx provides comparable information to the more invasive, ICP-based, PRx in the setting of elected ICP from high cephalic venous pressure. It is unknown why there is a slight improvement in the performance of PRx and HVx in increased ICP models over non-traumatic, hydrocephalic models that have been previously reported.
Ruesch et al. (2021)^45^	12 non-human primates (NHPs), *Macaca mulatta*, males with the age of 8.1 ± 1.7 years and weighing 9.9 ± 2.5 kg: • 7 NHPs were isoflurane-anesthetized • 5 NHPs were fentanyl-anesthetized	PRx	Induced ICP change by influencing pressure in a lumbar catheter, which was placed into the lateral ventricle in the brain, to get fluid to flow in the ventricle from the connected saline reservoir Introduced oscillatory perturbations in ICP at five frequencies. There were four oscillations performed per frequency, with the exception of eight oscillations for the fastest frequency to get an adequate time period for frequency analysis. ICP held at baseline (3–6 mm Hg) and then gradually increased up to 40 mm Hg after each set of ICP oscillations.	CBF was measured using a custom-built diffuse correlation spectroscopy system, and Lassen's curve was constructed based on the diffusion coefficient value. Lassen's curve was calculated for each NHP before they were averaged in groups of isoflurane and fentanyl anesthesia. The plateau in the Lassen's curve indicates intact cerebral autoregulation, and the sloped areas below and above the plateau are LLA and ULA, respectively.	Isoflurane-anesthetized NHPs showed values of PRx above zero at a broad range of CPP, with PRx >0.3 for the Lassen's curve plateau, which indicates cerebral autoregulation impairment. Fentanyl-anesthetized NHPs had an average negative trend, with PRx <0, in the Lassen's curve plateau, which indicates intact cerebral autoregulation. High ICP corresponds to low CPP, and during this time, cerebral autoregulation becomes impaired.
Zeiler et al. (2018a)^20^	Archived data of 12 New Zealand (NZ) rabbits were analyzed from previous studies.	PRx PAx Mx Sx Lx RAC	ICP was raised with Hartmann's solution into the lumbar cistern secondary to CSF infusion. Initially, ICP increased to reach a plateau of ∼40 mm Hg after ∼10 min, and then severe intracranial hypertension was produced by increasing the infusion rate, which corresponded to a mean ICP of 75 mm Hg.	LLA: • Plots of LDF-CBF vs. CPP or FVs vs. CPP were constructed using ICM+ software. • Breakpoint representing the LLA for each animal was identified at the intersection of two linear segments with the minimized sum residual squared error with the automated piece-wise linear regression conducted with R statistical software. • Mean LLA was determined by averaging all 12 LLA values obtained for the cohort of 12 rabbits.	Both PRx and PAx correlate with LLA and progressively become more positive below LLA. PRx threshold of zero does not represent the LLA, so it is unclear what physiological relevance it represents. RAC was not evaluated because it had poor performance compared to LLA and could not produce a reliable piece-wise model for RAC vs. CPP.

CBF, cerebral blood flow; COx, cerebral-oximetry index; CPP, cerebral perfusion pressure; CSF, cerebral spinal fluid; FVs, systolic flow velocity; HVx, hemoglobin volume index; ICM+, Intensive Care Monitoring software (Cambridge Enterprise Ltd, Cambridge, UK); ICP, intracranial pressure; LDF, laser Doppler flow; LDF-CBF, LDF-based CBF; LDx, laser Doppler index; LLA, lower limit of autoregulation; PRx, pressure-reactivity index; RAC, correlation between pulse amplitude of ICP and CPP; ULA, upper limit of autoregulation.

**FIG. 2.**
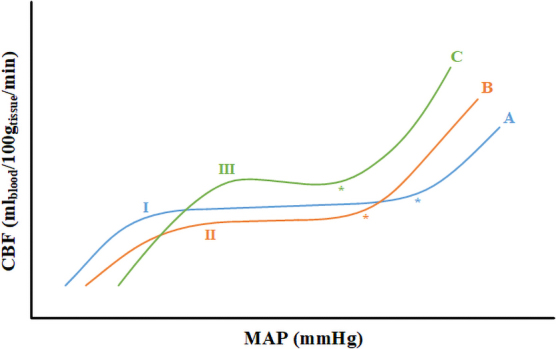
Variations of Lassen's autoregulatory curve observed in the studies. Curve A represents a normal autoregulatory curve^3,19,25,34–45^; curve B represents an autoregulatory curve during increased ICP^20,35,43^; and curve C represents an autoregulatory curve during hypercarbia.^44^ The LLA assessed is depicted in the figure by: “I” represents 14 studies^19,25,34–45^; “II” represents three studies^20,35,43^; and “III” represents one study.^44^ The asterisk (“*”) represents the ULA, which was not assessed in any study. CBF, cerebral blood flow; ICP, intracranial pressure; LLA, lower limit of autoregulation; MAP, mean arterial pressure; ULA, upper limit of autoregulation.

Figure 2 gives an illustration of variations in the autoregulatory curve noted in the studies along with indicating the LLA assessed in all the studies. Most of the studies measured multiple CVR indices and they include: six studies that measured cerebral-oximetry index (COx, correlation between regional cerebral oximetry [rSO_2_] and CPP),^25,34–38,40^ one measured COx-a (correlation between rSO_2_ and MAP),^36^ one measured wavelet COx (wCOx, correlation between wavelet phase shift in rSO_2_ and CPP),^25^ seven studies measured hemoglobin volume index (HVx, correlation between relative total hemoglobin and MAP),^25,38–41,43,44^ one measured wavelet HVx (wHVx, wavelet correlation between relative total hemoglobin and MAP),^25^ two measured LDx (correlation between laser-Doppler flux and MAP),^34,35^ one measured mean flow index (correlation between mean flow velocity and CPP),^20^ two measured pulse amplitude index (PAx, correlation between pulse amplitude of ICP),^19,20^ 10 studies measured pressure-reactivity index (PRx, correlation between ICP and MAP),^19,20,25,35,37,39,42–45^ one measured induced PRx (iPRx, correlation between ICP and MAP with induced variations in MAP),^37^ two measured wavelet PRx (wPRx, correlation between wavelet phase shift in ICP and CPP),^25,42^ two measured the correlation between pulse amplitude of ICP and CPP (RAC),^19,20^ one measured systolic flow index (correlation between systolic flow velocity and CPP),^20^ and one measured MAP-ICP phase shift (Δϕ_AI_).^37^

Eleven studies used neonatal swine^19,25,34–42^ as their animal model whereas two studies used juvenile domestic pigs,^43,44^ one study used rhesus macaque,^45^ and one study used New Zealand rabbits.^20^

### Hypotension/hypertension perturbation

Most of the studies that induced hypotension did it by gradually inflating the balloon catheter in the inferior vena cava (Table 1),^19,25,34–36,38–41^ except for one where hypotension occurred with the gradual hemorrhage by syringe-pump withdrawal at a calculated rate of blood volume per hour.^37^ From the 10 studies that induced hypotension, only three of them also induced hypertension by slowly inflating an aortic balloon catheter.^38,40,41^ Overall, all the CVR indices that were measured in the hypotension model were able to distinguish whether CPP was above or below the LLA, and these indices included: COx,^25,34–36,38,40,41^ COx-a,^36^ wCOx,^25^ HVx,^25,38–41^ wHVx,^25^ LDx,^34,35^ PAx,^19^ PRx,^19,25,35,37,39^ iPRx,^37^ wPRx,^25^ RAC,^19^ and Δϕ_AI_.^37^

Results from studies show that during hypotension, PRx, COx, LDx, and HVx accurately detected CPP/MAP above and below LLA.^25,34,35,38–41^ One study mentioned that LDx performed better than COx, and the agreement between these indices was limited but greatly improved with averaging values.^34^ The increase in PRx to more positive values (denoting impaired CVR) had a blunted response at CPP values around LLA, when compared with autoregulation curves derived from COx and LDx.^35^

In corollary, one study had shown that HVx is an excellent alternative to PRx.^39^ In the same animal model, it has been shown that MAP-derived COx has an accuracy that is very much comparable to CPP-based COx, with COx-a having a slightly higher threshold for discriminating the LLA as compared to COx.^36^

Variants of PRx displayed varying performance with regard to their availability measure aspects of the Lassen autoregulatory curve. During hypotension with mean pressures below LLA, the iPRx and Δϕ_AI_ were significantly lower as compared to normotension and hypotension above LLA where both iPRx and Δϕ_AI_ were not different.^37^ Wavelet indices (wPRx, wCOx, and wHVx) did not increase as much as their corresponding correlation indices (PRx, COx, and HVx) did as MAP decreased below LLA. Also, all wavelet indices had lower variability than their correlation index counterparts given that the wavelet indices used a methodology to decrease signal noise.^25^

Finally, non-PRx ICP-derived CVR indices were only sparingly mentioned. Strong conclusions about PAx and RAC cannot be made given that only one study evaluated them against PRx with a small number of animals, but the preliminary evidence shows that both of these indices validate against LLA within a model of hypotension whereas PRx seemed to be superior.^19^

The results from the handful of studies that induced hypertension showed that the values of CVR indices measured (COx, HVx) were consistent with preserved autoregulation.^38,40,41^ Of note, these studies did not successfully evaluate the ULA. Subsequently, we cannot comment on the ability of any of the described indices' ability to estimate aspects of the ULA at this time.

### Intracranial pressure perturbation

There were only four studies on ICP perturbation, as seen in Table 2, and they induced ICP change using various methods, including: cerebrospinal fluid (CSF) infusion,^20,35^ gradual inflation of a latex balloon catheter in the superior vena cava,^43^ and influencing fluid to flow from a saline reservoir to lateral ventricle in the brain by lumbar catheter.^45^ Under both naïve and elevated ICP conditions, similar values were obtained above LLA using PRx, COx, HVx, and LDx,^35,43^ but two studies were able to relate high ICP to low CPP where CA becomes impaired^20,45^ and both PRx and PAx correlated well with LLA.^20^

### Perturbation in pathological states

Other methods of inducing perturbation in the studies included cardiac arrest,^41^ cardiac arrest with hypothermia,^25,38,40^ hypercarbia,^44^ and positive end-expiratory pressure (PEEP) oscillations as seen in Table 3.^37,42,45^ Cardiac arrest was induced by hypoxic-asphyxic in all the related studies^25,38,40,41^ whereas hypothermia was induced by cooling the temperature for a period of time or multiple short time periods.^25,38,40^ PRx, wPRx, COx, wCOx, HVx, and wHVx indices were able to discriminate CPP/MAP above and below the LLA in cardiac arrest and hypothermia,^25,38,40^ but it should be noted that the CPP/MAP change had to be driven to evaluate LLA taking the form of arterial hypotension or ICP increase in a brain-injured state attributable to cardiac arrest or hypothermia. One study reported that the accuracy of COx and HVx was slightly less in post-cardiac arrest animals.^40^

**Table 3.** Performance of Continuous Cerebrovascular Reactivity Indices: “Other” Studies (Cardiac Arrest, Hypothermia, etc.)

References	Animal model details	CVR indices measured	Method of inducing perturbation	Aspects of the Lassen autoregulatory curve measured	Conclusions regarding continuous indices
*Cardiac arrest*					
Lee et al. (2012)^41^	Neonatal male piglets were 3–5 days old and weighing 1.0–2.5 kg. To evaluate LLA, 24 piglets were divided into three groups (8 per group): • 1 day of recovery after arrest • 2 days of recovery after arrest • 2 days of recovery after the sham procedure To evaluate autoregulatory response to hypertension, a separate cohort of 10 piglets were divided into two groups (5 per group): • 2 days of recovery after arrest • 2 days of recovery after the sham procedure For neurobehavioral testing and histology, a separate cohort of 11 piglets were put into two groups: • Underwent arrest (6 piglets) • Sham surgeries without cranial instrumentation or blood pressure manipulation (5 piglets)	COx HVx	HA cardiac arrest	LLA: • LDF plotted as a function of CPP. • LLA is defined as the CPP at the intersection of two regression lines resulting in the lowest combined error squared.	COx and HVx were good predictors of whether CPP was above or below the LLA. During hypotension, COx and HVx increased as CPP was decreased, with the slope of this relationship being less than zero. • For piglets in the group of 1 day of recovery after arrest, the relationship for COx was biphasic, with the slope below the LLA significantly more negative than the slope above the LLA (*p* = 0.014). During hypertension, the autoregulatory function measured by sROR was similar between piglets that recovered for 2 days after sham surgery and piglets that recovered for 2 days after resuscitation from HA cardiac arrest.
*Cardiac arrest and hypothermia*					
Larson et al. (2013)^38^	Neonatal male piglets were 3–5 days old and weighing 1.0–2.5 kg. 48 piglets were randomized into four groups, each containing 12 piglets: • HA injury with hypothermia • HA injury with hypothermia and rewarming • Sham surgery with hypothermia • Sham surgery with hypothermia and rewarming Hypotension was induced in half of the piglets (6) in each of the four groups, and hypertension was induced in the other half of the piglets (6).	COx HVx	• HA cardiac arrest with hypothermia • HA cardiac arrest with hypothermia and rewarming • Sham surgery with hypothermia • Sham surgery with hypothermia and rewarming	LLA: • LDF plotted as a function of CPP. • CPP at the intersection of two regression lines with the lowest combined residual squared error was defined as the LLA.	COx (*p* > 0.10) and HVx (*p* > 0.05) accurately detected differentiated CPP below and above the LLA. Both HVx and COx increased as CPP decreased. During hypertension, cerebral autoregulation remained functional after HA injury after hypothermia or hypothermia and rewarming.
Lee et al. (2011)^40^	64 neonatal male swine were 3–5 days old and weighing 1.0–2.5 kg. Piglets were divided into eight groups: • Hypotensive cohorts ○ Post-arrest normothermia ○ Post-arrest hypothermia ○ Sham normothermia ○ Sham hypothermia • Hypertensive cohorts ○ Post-arrest normothermia ○ Post-arrest hypothermia ○ Sham normothermia ○ Sham hypothermia	COx HVx	Hypotensive cohorts: • Post-HA cardiac arrest normothermia • Post-HA cardiac arrest hypothermia • Sham normothermia • Sham hypothermia Hypertensive cohorts: • Post-HA cardiac arrest normothermia • Post-HA cardiac arrest hypothermia • Sham normothermia • Sham hypothermia	LLA: • LDF plotted as a function of CPP • LLA is defined as the CPP at the intersection of two linear regression lines resulting in the lowest combined error squared.	HVx and COx were good predictors of whether CPP was above or below the LLA, but the accuracy was slightly less in post-arrest animals. In hypotension cohorts, sham animals with CPP below LLA were detected by COx 0.41 and HVx 0.22 whereas arrested animals with CPP below LLA were detected by COx 0.42 and HVx 0.21. There was a 50% probability that CPP exceeded LLA at the HVx and COx values given above. In hypertension cohorts, as CPP increased, HVx and COx values did not increase above zero and were consistent with preserved autoregulation during post-resuscitation normothermia and hypothermia.
Liu et al. (2020)^25^	Data from 68 neonatal piglets were analyzed from three previous studies where 35 piglets were resuscitated from cardiac arrest and 33 were sham piglets.	PRx wPRx COx wCOx HVx wHVx	Reanalyzed data from three published piglet studies of HA cardiac arrest and hypothermia: • First study had piglets randomized to cardiac arrest or sham procedure followed by a 6-h recovery period with whole-body hypo- or normothermia. • Second study had piglets randomized to cardiac arrest or sham procedure followed by overnight hypothermia with or without rewarming. • Third study had piglets randomized to cardiac arrest or sham procedure with 1 or 2 days of normothermic recovery.	LLA: • Each piglet's MAP at LLA was identified by ICM+ software, which uses piece-wise regression to dichotomize the data and fit two linear regression lines with the lowest combined error squared. • Each piglet's laser Doppler flow-derived LLA was considered to be the gold standard to test whether autoregulation indices identified MAP above or below the LLA.	All indices discriminated MAP above and below the LLA (*p* < 0.001). Distinguishing MAP above from below LLA did not differ between COx and wCOx or between PRx and wPRx whereas HVx had a significantly higher discriminatory value than wHVx in detecting dysfunctional autoregulation. Wavelet indices highly correlated with their respective correlation indices (*r* = 0.78, *p* < 0.001 for PRx and wPRx; *r* = 0.66, *p* < 0.001 for COx and wCOx; *r* = 0.78, *p* = 0.002 for HVx and wHVx;). All the correlation indices became positive and approached +1 as blood pressure deviated farther below the LLA during induced hypotension. Wavelet indices increased as MAP decreased below LLA, but not as much as that observed with the correlation indices. Mean values for all indices were each significantly lower when MAP was above LLA than below in paired comparisons (*p* < 0.001 for each index). All wavelet indices had lower variability than their corresponding correlation index. The wavelet methodology uses a coherence threshold to eliminate poorly coherent signals to decrease signal noise.
*Hypercarbia*					
Nusbaum et al. (2016)^44^	Juvenile domestic pigs were divided into two groups: • Normocarbia group (control group) contained 10 pigs. • Hypercarbia group (high-CO_2_ group) contained 8 pigs.	• PRx: based on slow-wave changes in measured ICP taken from an invasive ventricular catheter • HVx: NIRS-based index	After completion of baseline measurements in the hypercarbia group, lasting 1 h, an external CO_2_ cylinder was used to bleed CO_2_ into the inspired gas mixture to maintain end-tidal CO_2_ at 20 mm Hg above baseline for 1 h. Then, end-tidal CO_2_ was maintained at 40 mm Hg above baseline for 1 h to achieve steady-state arterial pCO_2_ >80 Torr. Control piglets were maintained at baseline normocarbic levels for 3 h. After a 3-h testing period, all subjects underwent controlled hemorrhage by femoral venous catheter, which slowly decreased CPP to ∼10 mm Hg. This reduced the MAP over 1–2 h, producing a quasi-steady-state CPP with spontaneous slow fluctuations.	LLA: • A scatterplot of 60-sec-averaged values of LDF vs. CPP was made using the ICM+ software for each piglet. • CPP at the intersection of two regression lines with the lowest combined residual squared error was defined as the autoregulatory breakpoint for each animal.	Despite the presence of hypercarbia, PRx and HVx accurately detected the LLA. Elevated CO_2_ did not interfere with NIRS-based methods. Laser Doppler-based estimates of LLA had similar increases with CO_2_ exposure as NIRS-based measurements.
*PEEP oscillations*					
Brady et al. (2012)^37^	10 neonatal swine	Without PEEP oscillation: • PRx With PEEP oscillation: • iPRx • Δϕ_AI_	Induced variations in MAP using PEEP modulation. • PEEP oscillated between 5 and 10 cmH_2_O in a sine-wave pattern with a period of 60 sec.	LLA: • Continuous cortical laser Doppler flux was used to delineate the LLA. • Flux measurements were plotted across CPP to determine LLA by piece-wise linear regression at the intersection of two best-fit lines with the lowest residual error squared.	PEEP modulation significantly improved the precision of PRx monitoring. At normal MAP: • Before PEEP oscillations, the resultant PRx was −0.06. • With PEEP oscillations: ○ iPRx at normotension was −0.39, and at hypotension above LLA was −0.42 while below LLA was 0.32 (*p* = 0.0004). ○ Δϕ_AI_ at normotension was 150 degrees, and at hypotension above LLA was 161 degrees while below LLA was −31 degrees (*p* < 0.0001). For CPP below LLA: • iPRx threshold value of −0.04 was both 95% sensitive and 95% specific. • Δϕ_AI_ had a phase-angle difference of <115 degrees and was 95% sensitive and <103 degrees was 95% specific. Δϕ_AI_ was significantly lower, causing significantly lower iPRx, during hypotension below LLA compared to the normal pre-load state and mild hypotension where iPRx and Δϕ_AI_ were not different.
Liu et al. (2018)^42^	Two separate piglet models of domestic swine 1–2 days of age and weighing 1–5 kg were analyzed from previous studies: • PEEP group contained 12 piglets. • Non-PEEP group contained 17 piglets: 10 in the naïve ICP (10 mm Hg) group and 7 in the elevated ICP (20 mm Hg) group.	PRx wPRx	PEEP group: regular, sinusoidal (1-min period), strong MAP oscillations were induced using modulated PEEP. Non-PEEP group: spontaneous MAP waves at two ICP levels (naïve and elevated)	LLA: • A scatterplot of 1-min-averaged LDF vs. CPP was generated for each piglet. • CPP at the left intersection of two lines defined by a piece-wise linear regression model was defined as the LLA.	wPRx produced a more stable result than PRx and distinguished CPP above and below LLA more significantly when spontaneous MAP and ICP waves were analyzed. In the PEEP group: • PRx (*p* < 0.001) and wPRx (*p* < 0.001) were increased significantly whereas CPP was decreased below LLA, indicating worse autoregulation. • AUC-ROC curve showed that both wPRx and PRx can differentiate CPP above or below LLA. In the non-PEEP group: • wPRx (*p* = 0.003) showed a more significant increase than PRx (*p* = 0.04). • AUC-ROC curve showed that both wPRx and PRx can differentiate CPP above or below LLA.
Ruesch et al. (2021)^45^	12 non-human primates (NHPs), *Macaca mulatta*, males with the age of 8.1 ± 1.7 years and weighing 9.9 ± 2.5 kg: • 7 NHPs were isoflurane-anesthetized • 5 NHPs were fentanyl-anesthetized	PRx	Oscillation induced in MAP by oscillating PEEP at same frequencies as ICP with baseline value set to 6 cmH_2_O (8.1 mm Hg) with a magnitude of 4 cmH_2_O (5.4 mm Hg). MAP oscillations were performed after ICP oscillations on every ICP baseline in the same order of frequencies.	CBF measured using a custom-built diffuse correlation spectroscopy system and Lassen's curve was constructed based on the diffusion coefficient value. Lassen's curve calculated for each NHP before they were averaged in groups of isoflurane and fentanyl anesthesia. The plateau in the Lassen's curve indicates intact cerebral autoregulation, and the sloped areas below and above the plateau are LLA and ULA, respectively.	Isoflurane-anesthetized NHPs showed values of PRx above zero at a broad range of CPP, with PRx >0.3 for the Lassen's curve plateau, which indicates cerebral autoregulation impairment. Fentanyl-anesthetized NHPs had an average negative trend, with PRx <0, in the Lassen's curve plateau, indicating intact cerebral autoregulation. High ICP corresponds to low CPP, and during this time, cerebral autoregulation becomes impaired.

AUC-ROC, area under receiver-operator characteristic curve; COx, cerebral-oximetry index; CPP, cerebral perfusion pressure; HA, hypoxic-asphyxic; HVx, hemoglobin volume index; ICM+, Intensive Care Monitoring software (Cambridge Enterprise Ltd, Cambridge, UK); ICP, intracranial pressure; iPRx, induced PRx; LDF, laser Doppler flow; LDx, laser Doppler index; LLA, lower limit of autoregulation; MAP, mean arterial pressure; NIRS, near-infrared spectroscopy; pCO_2_, partial pressure of CO_2_; PEEP, positive end-expiratory pressure; PRx, pressure-reactivity index; ROC, receiver operator characteristic; ULA, upper limit of autoregulation; wCOx, wavelet COx; wHVx, wavelet HVx; wPRx, wavelet PRx; Δϕ_AI_, MAP-ICP phase shift.

There was only one study on hypercarbia, and CO_2_ levels were elevated by bleeding CO_2_ from a cylinder into the gas mixture inspired by the piglets. Despite the presence of hypercarbia, PRx and HVx were able to accurately detect the LLA, and the elevated CO_2_ did not interfere with the NIRS readings.^44^

Finally, PRx variants were evaluated during PEEP modulation. With PEEP modulation, oscillations in MAP were induced^37,42,45^ and the precision of iPRx was improved compared to PRx.^37^ The wPRx produced a more stable result than PRx while distinguishing CPP above and below the LLA. However, both indices increased significantly while CPP was decreased below LLA in the PEEP group.^42^

## Discussion

Through comprehensive evaluation of the pre-clinical literature surrounding continuous CVR measures and their ability to measure aspects of the autoregulation curve, some interesting findings deserve highlighting.

First, the available literature lacked the inclusion of most of the CVR metrics of interest, provided in Supplementary Appendix SB. Thus, we can only comment on a select number of metrics based on ICP, NIRS, and TCD (to a limited extent). The handful of metrics evaluated by the eligible literature show that they can accurately detect CPP/MAP above the LLA and below the LLA. Thus, based on the available literature, end-users of such continuous measures can take some comfort in knowing that the listed metrics can estimate the LLA. However, all of the studies looked at the relationship between the continuous metrics and the LLA aspect of the Lassen autoregulatory curve, but none of the studies looked at relating the continuous metrics to the ULA aspect of the Lassen autoregulatory curve. Even though only one study could fully reconstruct the Lassen autoregulatory curve with LLA and ULA, it had only associated the continuous metrics to the intact autoregulation region of the curve.^45^ Traditionally, the region between LLA and ULA has been described as a plateau, but emerging evidence suggests that the intact autoregulation region of the curve is not a plateau.^46,47^

The study by Ruesch and colleagues^45^ showed that fentanyl-anesthetized non-human primates had an averaged negative trend between the LLA and ULA, which had been generally regarded as the plateau. This highlights the need for future validation work for both the LLA and new studies to evaluate the ULA along with exploring the intact autoregulation region, particularly in different large animals given that responses may vary. Similarly, with the ever-increasing number of metrics emerging based on continuous multi-modal monitoring of cerebral physiology, new pre-clinical studies encompassing all multi-modal metrics concurrently while evaluating the LLA and ULA are required to get a better perspective on their ability to estimate the Lassen autoregulatory curve.

Second, regarding the NIRS-based potentially non-invasive metrics, some important aspects regarding their ability to discern the LLA were noted. A study comparing COx with LDx during arterial hypotension concluded that LDx performed better than COx^34^; however, this should not be a surprise given that LDx is derived from LDF and the LLA was defined at the intersection of two regression lines on the LDF versus CPP scatterplot. The results from studies show that during hypotension, all the CVR metrics evaluated were able to detect CPP/MAP above and below LLA accurately. Among these studies, the commonly evaluated CVR indices were COx, PRx, and HVx, where HVx was mentioned to be an excellent alternative to PRx^39^ and COx derived from either MAP or CPP had comparable accuracy.^36^ Also, it has been shown that the wavelet counterparts of the three commonly evaluated indices had lower variability attributable to decreased signal noise.^25^

Of the 10 studies categorized as arterial hypotension/hypertension, only three of them induced hypertension along with hypotension^38,40,41^ whereas the rest only induced hypotension.^19,25,34–37,39^ The results from the hypertension studies showed that autoregulation was preserved, as indicated from COx and HVx values,^38,40,41^ but, as mentioned above, evaluation of these CVR indices to estimate aspects of ULA cannot be commented on because ULA was not successfully evaluated.

Third, with respect to different ICP-derived measures, preliminary evidence shows that the newer ICP-derived CVR indices, PAx and RAC, can estimate the LLA.^19^ However, PRx appears to remain superior in measuring the LLA in arterial hypotension models.^19^ Similarly, PAx and PRx correlated well with LLA during intracranial hypertension, where RAC performed poorly.^20^ Two other studies obtained similar values above LLA using PRx, COx, HVx, and LDx under both naïve and elevated ICP conditions.^35,43^ Thus, despite some literature demonstrating the ability of PAx and RAC to estimate aspects of the LLA, their exact role beyond PRx in TBI monitoring remains unclear.

Fourth, some studies used other models of inducing perturbation where specific pathological states were studied; common models were cardiac arrest with hypothermia and PEEP oscillations to ICP and/or MAP. PRx, COx, and HVx, along with their wavelet counterparts, accurately discriminated CPP/MAP above and below LLA in the model of cardiac arrest and hypothermia.^25,38,40,41^ With PEEP oscillations to modulate MAP, iPRx and wPRx gave improved and stable results of distinguishing CPP above and below LLA as compared to PRx.^37,42^ There was only one study on hypercarbia where elevated CO_2_ did not affect the ability of PRx and HVx to detect the LLA during gradual arterial hypotension induced by continuous hemorrhage.^44^ Thus, it appears that the ability of PRx (and its variants), COx, and HVx to estimate the LLA is unaffected by the pathological state or co-manipulations of systemic physiology. Hence, it was important to review the pre-clinical literature given that such findings offer some confidence in their ability to perform as continuous measures at the bedside for TBI patients.

Finally, most of the articles reviewed looked at static aspects of CA. Static autoregulation measures refer to those metrics that are derived when both aspects of physiological measures (i.e., surrogate for pulsatile CBF/CBV and driving pressure for flow) have reached a steady state, where autoregulatory responses are assessed over a longer time frame of minutes to hours.^46^ This is in contrast to dynamic autoregulation, where autoregulatory capacity is assessed in a much shorter temporal scale, on the order of seconds to minutes, in response to rapid changes in a driving pressure to flow.^46^ However, despite most of the articles having evaluated static aspects of CA and some fundamental physiological differences between the techniques, the findings here carry potential importance for both static and dynamic autoregulation measurement techniques. There is evidence that points to the general agreement of static and dynamic aspects of CA in impaired autoregulation under challenges in ICP and blood pressure.^34,45,48^ In normal anesthetized adults with intact or impaired autoregulation,^48^ and in non-human primates under anesthesia-induced autoregulation impairment,^45^ it has been demonstrated that there is a good correlation between both techniques to assess CA, although dynamic CA seems to be initially more reduced than static CA. Such an agreement between static and dynamic CA would be consistent given that the more momentary physiological factors assessed by dynamic CA would be sufficiently identical to the longer methods of assessment in static CA. However, in situations where paroxysmal physiological responses occur, static autoregulation may be unaffected and thus a disagreement between dynamic and static CA assessments. This enforces the idea that determination of CA must be validated through continuous methods.

### Limitations

Despite the interesting findings outlined in this scoping review, there are some significant limitations that deserve highlighting. First, the uncovered literature was very heterogeneous in design, which means that there was limited ability to cross-sectionally evaluate the relationship between studies based on various combinations of CVR indices used in each method of perturbation. Further to this, the different animal models and species utilized limit the comparability between similar indices and perturbation results. Second, most studies used a small number of animals in their model design. This is likely secondary to the cost of such models and experiments. Such small numbers limit the definitiveness of the findings outlined and emphasize the need for further validation studies. Third, some models described used neonatal animals. This raises concerns of cerebrovascular immaturity and translatability of findings to adult animals or humans. Fourth, from the numerous multi-modal cerebral physiological monitoring devices noted in bedside monitoring to derive continuous CVR indices,^19,20^ our review highlights that only a few have been studied pre-clinically. Thus, we cannot definitively indicate the utility of TCD, PbtO_2_, and Hemedex™ CBF-based techniques in their ability to measure the autoregulatory curve.

Fifth, we were unable to find literature documenting the ability of such continuous CVR metrics to estimate the ULA, which could be attributable to the occurrence of heart failure at higher CPP as mentioned by a couple of studies. As such, at this time, it is unknown whether such a metrics measure can discriminate more than just the LLA. Finally, and most important, the results from studies with animal models do not directly translate to human patients, though preliminary findings in humans support their ability to estimate the LLA.^49,50^

### Future directions

Moving forward, there are some important areas for future research. First, validation of the above findings is required, given that most are based on single-institution, single-study findings in a limited number of animals per study. Such work will require specialized centers for large animal research, dedicated to precision medicine in neural injury. These large animal models would be best positioned to facilitate translatability to adult human neurocritical care. Second, studying the performance of such indices in both healthy- and injury-state models is key to be able to differentiate between intact and dysfunctional autoregulation using each of the indices. Given that different neuropathological states may cause different changes to the shape and nature of the autoregulatory curve, having control pre-clinical models in the healthy state are critical to our understanding of fundamental changes in cerebral autoregulation that may occur in disease.

Further, in providing validation for a continuous surrogate measure of CA, we must have the utmost confidence in their ability to estimate various aspects of autoregulation during both the healthy and diseased states before they are adopted into routine clinical-care provision. This requires, again, specialized center expertise in model development, multi-modal cerebral physiological monitoring, and biomedical engineering capability. Such models would benefit from having both focal and diffuse injury patterns, including those modeling stroke, TBI, and anoxic brain injury. Third, work here would be bolstered by evaluating differences between both neonatal and adult models. Existing literature supports the use of pig models to facilitate such work.^51^ Fourth, integrating physiological monitoring with histopathological correlations could improve our fundamental understanding of tissue consequences of impaired CA, based on different monitoring modalities, across different model ages and across the spectrum of neuropathological states. Finally, such large animal-model platforms would provide the ideal setting to explore directed therapeutics aimed at prevention and treatment of impaired CA, facilitating the needed pre-clinical investigation of precision therapeutics before application in humans.

## Conclusion

This literature demonstrates that most of the measured indices (COx, COx-a, wCOx, HVx, wHVx, LDx, PAx, PRx, iPRx, wPRx, RAC, and Δϕ_AI_) showed the ability to distinguish CPP/MAP from above and below the LLA with various methods of perturbation in animal models. However, most studies focused on a small number of animal populations, with only a few simulating ICP elevations observed in TBI, so the conclusions drawn from these studies should be taken with caution. Also, none of the indices evaluated the ULA aspect of the Lassen autoregulatory curve. Therefore, further research is required to fully assess the relationship between both the LLA and ULA aspects of the Lassen autoregulatory curve to CVR indices in a larger pre-clinical population size.

## Supplementary Material

Supplemental data

Supplemental data

Supplemental data

Supplemental data

## Supplementary Material

Supplementary Appendix SA

Supplementary Appendix SB

Supplementary Appendix SC

Supplementary Appendix SD

## Abbreviations Used

CA: cerebral autoregulation
CBF: cerebral blood flow
CBV: cerebral blood volume
COx: cerebral-oximetry index
CPP: cerebral perfusion pressure
CSF: cerebrospinal fluid
CVR: cerebrovascular reactivity
HVx: hemoglobin volume index
ICP: intracranial pressure
iPRx: induced PRx
LDF: laser Doppler flowmetry
LDx: laser Doppler index
LLA: lower limit of autoregulation
MAP: mean arterial pressure
NIRS: near-infrared spectroscopy
PAx: pulse amplitude index
PbthO_2_: brain tissue oxygen
PEEP: positive end-expiratory pressure
PRISMA: Preferred Reporting Items for Systematic Reviews and Meta-Analysis
PRx: pressure-reactivity index
rSO_2_: regional cerebral oximetry
TBI: traumatic brain injury
TCD: transcranial Doppler
TDF: thermal diffusion flowmetry
ULA: upper limit of autoregulation
wCOx: wavelet COx
wHVx: wavelet HVx
wPRx: wavelet PRx
