# Supplementary material for: Continuous Time-Domain Cerebrovascular Reactivity Metrics and Discriminate Capacity for the Upper and Lower Limits of Autoregulation: A Scoping Review of the Animal Literature
Source: Neurotrauma Rep. 2021 Dec 20;2(1):639–59. doi: 10.1089/neur.2021.0043 (PMC8742280; doi:10.1089/neur.2021.0043)
Supplement: Supplemental data [file Supp_AppB.docx]

Appendix B – CVR Metrics of Interest:

| **Abbreviation** | **CVR Metric Description** |
| --- | --- |
| CBFx | Cerebral Blood Flow Index – correlation between CBF and CPP |
| CBFx-a | Cerebral Blood Flow Index – correlation between CBF and MAP |
| COx | Cerebral Oximetry Index – correlation between rSO_2_ and CPP |
| COx-a | Cerebral Oximetry Index – correlation between rSO_2_ and MAP |
| Dx | Diastolic Flow Index – correlation between FVd and CPP |
| Dx-a | Diastolic Flow Index – correlation between FVd and MAP |
| Hbx | Deoxyhemoglobin Index – correlation between Hb and CPP |
| Hbx-a | Deoxyhemoglobin Index – correlation between Hb and MAP |
| HbOx | Oxyhemoglobin Index – correlation between HHb and CPP |
| HbOx-a | Oxyhemoglobin Index – correlation between HHb and MAP |
| HVx | Hemoglobin Volume Index – correlation between relative total hemoglobin and MAP |
| iPRx | Induced Pressure Reactivity Index – correlation between ICP and MAP with induced variations in MAP |
| L-PRx | Long Pressure Reactivity Index – 20 mins correlation between ICP and MAP |
| LAx | Low-Frequency Autoregulation Index – minute-by-minute correlation between ICP and CPP |
| LDx | Laser-Doppler Index – correlation between laser-Doppler flux and MAP |
| Lx | Correlation between LDF-based CBF and CPP |
| Lx-a | Correlation between LDF-based CBF and MAP |
| Mx | Mean Flow Index – correlation between FVm and CPP |
| Mx-a | Mean Flow Index – correlation between FVm and MAP |
| ORx | Oxygen Reactivity Index – correlation between PbtO_2_ and CPP |
| PAx | Pulse Amplitude Index – correlation between AMP and MAP |
| PRx | Pressure Reactivity Index – correlation between ICP and MAP |
| PRx55-15 | Pressure Reactivity Index – correlation between bandpass filtered ICP and MAP |
| RAC | Correlation between pulse amplitude of ICP and CPP |
| Sx | Systolic Flow Index – correlation between FVs and CPP |
| Sx-a | Systolic Flow Index – correlation between FVs and MAP |
| THx | Total Hemoglobin Index – correlation between THI and CPP |
| THx-a | Total Hemoglobin Index – correlation between THI and MAP |
| TOx | Tissue Oxygen Index – correlation between TOI and CPP |
| TOx-a | Tissue Oxygen Index – correlation between TOI and MAP |
| wCOx | Wavelet Cerebral Oximetry Index – correlation between wavelet phase shift in rSO_2_ and CPP |
| wHVx | Wavelet Hemoglobin Volume Index – correlation between relative total hemoglobin and MAP |
| wPRx | Wavelet Pressure Reactivity Index – correlation between wavelet phase shift in ICP and CPP |
| AMP, pulse amplitude of ICP; CBF, cerebral blood flow; CPP, cerebral perfusion pressure; FVd, diastolic flow velocity; FVm, mean flow velocity; FVs, systolic flow velocity; Hb, deoxyhemoglobin; HbO, oxyhemoglobin; ICP, intracranial pressure; LDF, laser-doppler flow; MAP, mean arterial pressure; PbtO_2_, brain tissue oxygenation; rSO_2_, regional cerebral oximetry; THI, total hemoglobin index; TOI, total oxygen index. | |
