# Supplementary material for: Continuous Time-Domain Cerebrovascular Reactivity Metrics and Discriminate Capacity for the Upper and Lower Limits of Autoregulation: A Scoping Review of the Animal Literature
Source: Neurotrauma Rep. 2021 Dec 20;2(1):639–59. doi: 10.1089/neur.2021.0043 (PMC8742280; doi:10.1089/neur.2021.0043)
Supplement: Supplemental data [file Supp_AppC.docx]

Appendix C – Database Searches

Embase, Global Health & MEDLINE:

| Embase <1974 to 2021 January 15> | | |
| --- | --- | --- |
| Global Health <1973 to 2021 Week 02> | | |
| Ovid MEDLINE(R) ALL <1946 to January 15, 2021> | | |
| 1 | Animal model.mp. [mp=ti, ab, hw, tn, ot, dm, mf, dv, kw, fx, dq, bt, id, cc, nm, kf, ox, px, rx, an, ui, sy] | 1536276 |
| 2 | animal.mp. [mp=ti, ab, hw, tn, ot, dm, mf, dv, kw, fx, dq, bt, id, cc, nm, kf, ox, px, rx, an, ui, sy] | 7280507 |
| 3 | experimental.mp. [mp=ti, ab, hw, tn, ot, dm, mf, dv, kw, fx, dq, bt, id, cc, nm, kf, ox, px, rx, an, ui, sy] | 2845823 |
| 4 | Experimental model.mp. [mp=ti, ab, hw, tn, ot, dm, mf, dv, kw, fx, dq, bt, id, cc, nm, kf, ox, px, rx, an, ui, sy] | 112637 |
| 5 | 1 or 2 or 3 or 4 | 9367165 |
| 6 | limit 5 to english | 8555388 |
| 7 | limit 6 to article | 7381798 |
| 8 | limit 7 to yr="1995 - 2021" | 5599201 |
| 9 | Cerebral blood flow.mp. [mp=ti, ab, hw, tn, ot, dm, mf, dv, kw, fx, dq, bt, id, cc, nm, kf, ox, px, rx, an, ui, sy] | 75661 |
| 10 | CBF.mp. [mp=ti, ab, hw, tn, ot, dm, mf, dv, kw, fx, dq, bt, id, cc, nm, kf, ox, px, rx, an, ui, sy] | 34590 |
| 11 | Cerebrovascular reactivity.mp. [mp=ti, ab, hw, tn, ot, dm, mf, dv, kw, fx, dq, bt, id, cc, nm, kf, ox, px, rx, an, ui, sy] | 3636 |
| 12 | Cerebral autoregulation.mp. [mp=ti, ab, hw, tn, ot, dm, mf, dv, kw, fx, dq, bt, id, cc, nm, kf, ox, px, rx, an, ui, sy] | 5422 |
| 13 | Autoregulation.mp. [mp=ti, ab, hw, tn, ot, dm, mf, dv, kw, fx, dq, bt, id, cc, nm, kf, ox, px, rx, an, ui, sy] | 34462 |
| 14 | Cerebral blood flow velocity.mp. [mp=ti, ab, hw, tn, ot, dm, mf, dv, kw, fx, dq, bt, id, cc, nm, kf, ox, px, rx, an, ui, sy] | 3940 |
| 15 | CBFV.mp. [mp=ti, ab, hw, tn, ot, dm, mf, dv, kw, fx, dq, bt, id, cc, nm, kf, ox, px, rx, an, ui, sy] | 1902 |
| 16 | 9 or 10 or 11 or 12 or 13 or 14 or 15 | 116974 |
| 17 | limit 16 to english | 107207 |
| 18 | limit 17 to article | 85848 |
| 19 | limit 18 to yr="1995 - 2021" | 64011 |
| 20 | Intracranial pressure.mp. [mp=ti, ab, hw, tn, ot, dm, mf, dv, kw, fx, dq, bt, id, cc, nm, kf, ox, px, rx, an, ui, sy] | 62003 |
| 21 | ICP.mp. [mp=ti, ab, hw, tn, ot, dm, mf, dv, kw, fx, dq, bt, id, cc, nm, kf, ox, px, rx, an, ui, sy] | 65348 |
| 22 | Transcranial Doppler.mp. [mp=ti, ab, hw, tn, ot, dm, mf, dv, kw, fx, dq, bt, id, cc, nm, kf, ox, px, rx, an, ui, sy] | 23451 |
| 23 | TCD.mp. [mp=ti, ab, hw, tn, ot, dm, mf, dv, kw, fx, dq, bt, id, cc, nm, kf, ox, px, rx, an, ui, sy] | 12576 |
| 24 | Near infrared spectroscopy.mp. [mp=ti, ab, hw, tn, ot, dm, mf, dv, kw, fx, dq, bt, id, cc, nm, kf, ox, px, rx, an, ui, sy] | 40228 |
| 25 | NIRS.mp. [mp=ti, ab, hw, tn, ot, dm, mf, dv, kw, fx, dq, bt, id, cc, nm, kf, ox, px, rx, an, ui, sy] | 16387 |
| 26 | Thermal diffusion.mp. [mp=ti, ab, hw, tn, ot, dm, mf, dv, kw, fx, dq, bt, id, cc, nm, kf, ox, px, rx, an, ui, sy] | 2197 |
| 27 | TDF.mp. [mp=ti, ab, hw, tn, ot, dm, mf, dv, kw, fx, dq, bt, id, cc, nm, kf, ox, px, rx, an, ui, sy] | 12339 |
| 28 | Laser Doppler.mp. [mp=ti, ab, hw, tn, ot, dm, mf, dv, kw, fx, dq, bt, id, cc, nm, kf, ox, px, rx, an, ui, sy] | 36279 |
| 29 | LDF.mp. [mp=ti, ab, hw, tn, ot, dm, mf, dv, kw, fx, dq, bt, id, cc, nm, kf, ox, px, rx, an, ui, sy] | 4482 |
| 30 | PbtO2.mp. [mp=ti, ab, hw, tn, ot, dm, mf, dv, kw, fx, dq, bt, id, cc, nm, kf, ox, px, rx, an, ui, sy] | 602 |
| 31 | Brain tissue oxygen.mp. [mp=ti, ab, hw, tn, ot, dm, mf, dv, kw, fx, dq, bt, id, cc, nm, kf, ox, px, rx, an, ui, sy] | 1322 |
| 32 | Licox.mp. [mp=ti, ab, hw, tn, ot, dm, mf, dv, kw, fx, dq, bt, id, cc, nm, kf, ox, px, rx, an, ui, sy] | 386 |
| 33 | 20 or 21 or 22 or 23 or 24 or 25 or 26 or 27 or 28 or 29 or 30 or 31 or 32 | 231857 |
| 34 | limit 33 to english | 210372 |
| 35 | limit 34 to article | 170693 |
| 36 | limit 35 to yr="1995 - 2021" | 149767 |
| 37 | 5 and 16 and 33 | 4774 |
| 38 | 8 and 19 and 36 | 3043 |
| 39 | limit 37 to english | 4514 |
| 40 | limit 39 to article | 3814 |
| 41 | limit 40 to yr="1995 - 2021" | 3043 |

BIOSIS:

| #1 | TS = (Animal model OR animal OR experimental OR Experimental model) | 8,733,563 |
| --- | --- | --- |
| #2 | TS = (Cerebral blood flow OR CBF OR Cerebrovascular reactivity OR Cerebral autoregulation OR Autoregulation OR Cerebral blood flow velocity OR CBFV) | 28,447 |
| #3 | TS = (Intracranial pressure OR ICP OR Transcranial Doppler OR TCD OR NIRS OR Near-Infrared Spectroscopy OR Thermal diffusion OR TDF OR Laser Doppler OR LDF OR PbtO2 OR Brain tissue oxygen OR Licox) | 62,115 |
| #4 | #1 AND #2 AND #3 | 5,306 |

Scopus:

| #1 | ALL(Animal model OR animal OR experimental OR Experimental model) AND DOCTYPE(ar) AND PUBYEAR > 1994 AND (LIMIT-TO (LANGUAGE,"English")) | 2,822,710 |
| --- | --- | --- |
| #2 | ALL(Cerebral blood flow OR CBF OR Cerebrovascular reactivity OR Cerebral autoregulation OR Autoregulation OR Cerebral blood flow velocity OR CBFV) AND DOCTYPE(ar) AND PUBYEAR > 1994 AND (LIMIT-TO (LANGUAGE,"English")) | 30,222 |
| #3 | ALL(Intracranial pressure OR ICP OR Transcranial Doppler OR TCD OR Autoregulation OR Near infrared spectroscopy OR NIRS OR Thermal Diffusion OR TDF OR Laser Doppler OR LDF OR PbtO2 OR Brain tissue oxygen OR Licox) AND DOCTYPE(ar) AND PUBYEAR > 1994 AND (LIMIT-TO (LANGUAGE,"English")) | 330 |
| #4 | #1 AND #2 AND #3  ( ALL ( animal AND model OR animal OR experimental OR experimental AND model ) AND DOCTYPE ( ar ) AND PUBYEAR > 1994 ) AND ( ALL ( cerebral AND blood AND flow OR cbf OR cerebrovascular AND reactivity OR cerebral AND autoregulation OR autoregulation OR cerebral AND blood AND flow AND velocity OR cbfv ) AND DOCTYPE ( ar ) AND PUBYEAR > 1994 ) AND ( ALL ( intracranial AND pressure OR icp OR transcranial AND doppler OR tcd OR autoregulation OR near AND infrared AND spectroscopy OR nirs OR thermal AND diffusion OR tdf OR laser AND doppler OR ldf OR pbto2 OR brain AND tissue AND oxygen OR licox ) AND DOCTYPE ( ar ) AND PUBYEAR > 1994 ) AND ( LIMIT-TO ( LANGUAGE , "English" ) ) | 36 |

Cochrane Library:

| #1 | (Animal model):ti,ab,kw OR (animal):ti,ab,kw OR (experimental):ti,ab,kw OR (Experimental model):ti,ab,kw | 87054 |
| --- | --- | --- |
| #2 | (Cerebral blood flow):ti,ab,kw OR (CBF):ti,ab,kw OR (Cerebral Circulation):ti,ab,kw OR (Brain Blood Flow):ti,ab,kw OR (Cerebral Perfusion Pressure):ti,ab,kw OR (Regional Cerebral Blood Flow):ti,ab,kw OR (Cerebrovascular reactivity):ti,ab,kw OR (Cerebral autoregulation):ti,ab,kw OR (Autoregulation):ti,ab,kw OR (Cerebral blood flow velocity):ti,ab,kw OR (CBFV):ti,ab,kw | 7114 |
| #3 | (Intracranial pressure):ti,ab,kw OR (ICP):ti,ab,kw OR (Subarachnoid Pressure):ti,ab,kw OR (Intracerebral Pressure):ti,ab,kw OR (Transcranial Doppler):ti,ab,kw OR (TCD):ti,ab,kw OR (Near infrared spectroscopy):ti,ab,kw (NIRS):ti,ab,kw OR (Near-Infrared Spectroscopy):ti,ab,kw OR (NIR Spectroscopy):ti,ab,kw OR (Thermal diffusion):ti,ab,kw OR (TDF):ti,ab,kw OR (Thermodiffusion):ti,ab,kw OR (Laser Doppler):ti,ab,kw OR (LDF):ti,ab,kw OR (PbtO2 ):ti,ab,kw OR (Brain tissue oxygen):ti,ab,kw OR (Licox):ti,ab,kw | 9964 |
| #4 | #1 AND #2 | 575 |
| #5 | #1 AND #2 AND #3 | 128 |
