# Supplementary material for: Continuous Time-Domain Cerebrovascular Reactivity Metrics and Discriminate Capacity for the Upper and Lower Limits of Autoregulation: A Scoping Review of the Animal Literature
Source: Neurotrauma Rep. 2021 Dec 20;2(1):639–59. doi: 10.1089/neur.2021.0043 (PMC8742280; doi:10.1089/neur.2021.0043)
Supplement: Supplemental data [file Supp_AppD.docx]

Appendix D – General Overview of Characteristics of Included Studies

| **References** | **Animal Model Details** | **CVR Indices Measured** | **Aspects of the Lassen Autoregulatory Curve Measured** | **Primary / Secondary Outcomes** | **Limitations** |
| --- | --- | --- | --- | --- | --- |
| Brady et al. (2007)^34^ | Piglets 3 to 8 days old and weighing 2.2 to 3.9 kg.  6 Piglets were made progressively hypotensive (low blood pressure) over 4-5 hours. | COx  LDx | LLA:   - A scatterplot of LDF vs CPP was made with all data for each piglet. - CPP at the intersection of 2 regression lines with the lowest combined residual squared error was defined as the autoregulatory breakpoint. | Primary: Test a newly derived index from time-domain analysis, COx, for detecting loss of autoregulatory vasoreactivity to spontaneous fluctuations in CPP.  Secondary: Test the new COx index against a similar but invasive method, LDx. | Using transient and spontaneous changes in MAP decreases the signal-to-noise ratio.  Dynamic decreases in cerebral O_2_ consumption could affect COx and LDx by giving a false appearance of intact autoregulation. |
| Brady et al. (2008)^35^ | Piglets were 5 to 10 days old and weighing 2.2 to 3.9 kg.  8 piglets were in the Naïve ICP group (ICP not altered)  6 piglets were in the Elevated ICP group (ICP altered to ≈20 mmHg)  Arterial hypotension: low blood pressure induced by a balloon catheter | PRx  COx  LDx | LLA:   - A scatterplot of 1-minute averaged LDF versus CPP was made for each piglet. - CPP at the intersection of 2 regression lines with the lowest combined residual squared error was defined as the autoregulatory breakpoint. | Primary: Evaluate three separate continuous monitors of autoregulation (LDx, COx and PRx).  Secondary: Determine if PRx accuracy dependent on intracranial compliance. | Individually, measurements of COx and LDx are subject to noise. |
| Brady et al. (2010)^36^ | Waveform recordings of 25 piglets 5 to 7 days old   - Identified and included from prior studies with naïve ICP along with intact recordings of ICP, MAP, red cell flux in parietal cortex using Laser Doppler and rSO_2_. | COx  COx-a | LLA:   - Continuous red cell flux, recorded from laser Doppler, was plotted as a function of MAP. - These plots were dischotomized to give 2 best fit lines having the lowest combined residual error squared and MAP at their intersection was defined as the LLA. - MAP at LLA was chosen as the gold standard instead of CPP at LLA since this study sought to evaluate if COx can detect MAP above and below LLA without an ICP monitor. | Primary: Compare the accuracy of MAP versus CPP using COx to measure autoregulation in a piglet model of arterial hypotension. | All data being obtained in infant swine with immature and uninjured brains limits the application of the data to adult populations. |
| Brady et al. (2012)^37^ | 10 Neonatal swine | PRx  iPRx  Δϕ_AI_ | LLA:   - Continuous cortical Laser Doppler Flux was used to delineate the LLA. - Flux measurements were plotted across CPP to determine LLA by piecewise linear regression at the intersection of 2 best fit lines with the lowest residual error squared. | Primary: Improve PRx precision by inducing variations in MAP using PEEP modulation (iPRx). | The study was not designed to detect the difference in accuracy among PRx, iPRx and Δϕ_AI_ but rather to determine the accuracy obtained with PEEP oscillation. |
| Larson et al. (2013)^38^ | Neonatal male piglets were 3 to 5 days old and weighing 1 to 2.5 kg.  48 piglets were randomized into four groups, each containing 12 piglets:   - HA injury with hypothermia - HA injury with hypothermia and rewarming - Sham surgery with hypothermia - Sham surgery with hypothermia and rewarming   Hypotension was induced in half of the piglets (6) in each of the four groups and hypertension was induced in the other half of the piglets (6). | COx  HVx | LLA:   - LDF plotted as a function of CPP. - CPP at the intersection of 2 regression lines with the lowest combined residual squared error was defined as the LLA. | Primary: Examine the effects of rapid rewarming from hypothermia on cerebral autoregulation in a neonatal swine model of HA brain injury.  Secondary: Determine if rewarming would impair cerebral autoregulation during hypertension compared with sustained hypothermia. | Normothermic sham and normothermic HA injury groups were not included because the study aimed to compare the effects of rewarming with hypothermia on cerebral autoregulation.  The study’s findings of intact cerebral autoregulation might not be generalizable to clinical situations since anesthesia provided is not commonly used in intensive care units and brain injuries can be more severe in clinical situations.  Sample sizes were small. |
| Lee et al. (2009)^39^ | 8 piglets were 5 to 10 days old and weighing 2.34 ± 0.47 kg.  Hypotension was induced in these piglets. | HVx  PRx | LLA:   - A scatterplot of 1-minute averaged LDF versus CPP was generated for each piglet. - CPP at the intersection of 2 regression lines with the lowest combined residual squared error was defined as the LLA. | Primary: Develop a new index of vascular reactivity, HVx, which is a moving linear correlation between slow waves of rTHb and MAP.  Secondary: Determine if HVx would be strongly correlated to PRx and HVx would accurately detect LLA in a swine model of induced hypotension. | Regional specificity of the HVx only describes vasculature in the reflective path of infrared light between the optodes of the NIRS-based monitor compared to PRx which uses a global measurement of ICP. The implications of this difference have not been determined and perhaps are situationally specific. |
| Lee et al. (2011)^40^ | 64 neonatal male swine were 3 to 5 days old and weighing 1 to 2.5 kg.  Piglets were divided into eight groups:   - Hypotensive cohorts   - Postarrest normothermia   - Postarrest hypothermia   - Sham normothermia   - Sham hypothermia - Hypertensive cohorts   - Postarrest normothermia   - Postarrest hypothermia   - Sham normothermia   - Sham hypothermia | COx  HVx | LLA:   - LDF plotted as a function of CPP. - LLA is defined as the CPP at the intersection of 2 linear regression lines resulting in the lowest combined error squared. | Primary: Determine the effects of HA cardiac arrest and moderate hypothermia on autoregulation in neonatal swine.  Secondary: Determine if HVx and COx accurately reflect autoregulation after HA cardiac arrest. | In the CPP range of 100-120 mmHg, a consistent increase in LDF was not observed in anesthetized piglets which could be due to anesthesia reducing the release of vasodilator prostanoids during hypertension or it may have extended the ULA by effects on baseline myogenic tone.  CPP was not increased above 100-120 mmHg because heart failure occurred.  These results in neonatal piglets may not apply to older children. |
| Lee et al. (2012)^41^ | Neonatal male piglets were 3 to 5 days old and weighing 1 to 2.5 kg.  To evaluate LLA, 24 piglets were divided into three groups (8 per group):   - One day of recovery after arrest - Two days of recovery after arrest - Two days of recovery after the sham procedure   To evaluate the autoregulatory response to hypertension, a separate cohort of 10 piglets were divided into two groups (5 per group):   - Two days of recovery after arrest - Two days of recovery after the sham procedure   For neurobehavioral testing and histology, a separate cohort of 11 piglets were put into two groups:   - Underwent arrest (6 piglets) - Sham surgeries without cranial instrumentation or blood pressure manipulation (5 piglets) | COx  HVx | LLA:   - LDF plotted as a function of CPP. - LLA is defined as the CPP at the intersection of 2 regression lines resulting in the lowest combined error squared. | Primary: Test the hypothesis that LLA would shift to a higher arterial blood pressure between 1 and 2 days of recovery after cardiac arrest and that the LLA would be detected by NIRS-derived indices of autoregulation in swine models of pediatric cardiac arrest.  Secondary: Test the hypothesis that autoregulation with hypertension would be impaired after cardiac arrest. | Unable to correlate loss of autoregulation with neurobehavioral deficits because autoregulation remained intact in the study.  A consistent increase in LDF was not observed in the CPP range of 100 to 135 mmHg which could be due to the small sample size.  CPP was not increased above 140 mmHg because heart failure occurred.  The robustness of COx and HVx could not be thoroughly evaluated because postarrest piglets did not have altered autoregulation.  The experimental design was unbalanced since a 1-day survival sham group was not included.  The 1-day recovery sham group and 1-day of recovery after arrest group were not examined in an effort to decrease the number of animals used since autoregulation was not expected to change from previous work.  It is unclear if the results in neonatal piglets apply to older children.  LDF measurement technique does not provide absolute flow units, which could differ after arrest. |
| Liu et al. (2018)^42^ | Two separate piglet models of domestic swine 1 to 2 days of age and weighing 1 to 5 kg were analyzed from previous studies:   - PEEP group contained 12 piglets. - Non-PEEP group contained 17 piglets: 10 in the naïve ICP (10 mmHg) group and 7 in the elevated ICP (20 mmHg) group. | PRx  wPRx | LLA:   - A scatterplot of 1-minute averaged LDF versus CPP was generated for each piglet. - CPP at the left intersection of 2 lines defined by a piecewise linear regression model was defined as the LLA. | Primary:   - Validate the wavelet method for cerebrovascular autoregulation assessment in conditions of high-power, regular, periodic waves of MAP. - Establish advantages and disadvantages of the wavelet method vs the well-established parameter, PRx. | The coherence threshold for the wavelet analysis was not calculated for each individual recording, but a single threshold was chosen to be calculated for the whole dataset to make the method applicable to real-time analysis. It should be noted that there may be considerable variability in the coherence threshold due to the differences in autocorrelation in individual datasets. |
| Liu et al. (2020)^25^ | - Data from 68 neonatal piglets were analyzed from three previous studies where 35 piglets were resuscitated from cardiac arrest and 33 were sham piglets. | PRx  wPRx  COx  wCOx  HVx  wHVx | LLA:   - Each piglet’s MAP at LLA was identified by ICM+ software which uses piecewise regression to dichotomize the data and fit two linear regression lines with the lowest combined error squared. - Each piglet’s laser Doppler flow-derived LLA was considered to be the “gold standard” to test whether autoregulation indices identified MAP above or below the LLA. | Primary: Compare wavelet and correlation metrics in the piglet model of cardiac arrest.  Secondary: Compare MAPopt values identified by wavelet to those from correlation metrics. | With the reanalyzed piglet cohorts from past studies, only male piglets were studied.  Piglets’ upper limit of autoregulation was not assessed because cardiopulmonary failure often occurs before this limit can be reached after cardiac arrest. |
| Nusbaum et al. (2014)^43^ | Juvenile domestic pigs were divided into two groups:   - The naïve ICP group contained 15 piglets with a mean weight of 63.1 kg. - The high ICP group contained 20 piglets with a mean weight of 54.2 kg. | - PRx: based on slow-wave changes in measured ICP taken from an invasive ventricular drain - HVx: NIRS-based index | LLA:   - A scatterplot of 60-second averaged values of LDF versus CPP was made using the ICM+ software for each piglet. - CPP at intersection of 2 regression lines with the lowest combined residual squared error was defined as the autoregulatory breakpoint for each animal. | Primary: Determine if ICP elevation from cerebral venous outflow obstruction would result in comparable alterations in the LLA. | The results from the obtained data of the juvenile piglets will not extrapolate to adult pig or human populations.  CBF measurement at a single discrete location in cortex can confound calculation of LLA if there are regional differences in CBF.  There is possibility that the slight decrease in average pH during high ICP period, most likely related to the infusion of intravenous saline solution, may have contributed to shifts in LLA but it is not likely based on previous studies.  There was a difference in baseline MAP and ICP between naïve and high ICP groups that can be partly explained by the significant difference in weight of the piglets between the two groups.  Animals in the high ICP group had dramatic differences in their LLA compared to those of the naïve group with comparable baseline MAP and ICP measurements but it is more likely that elevated ICP causes the increase in LLA.  In order to increase ICP beyond 20 mmHg, the model of inflating balloon catheter in the superior vena cava was used, which does not represent true fluid shifts cranially but is a valid model for obstructive processes. |
| Nusbaum et al. (2016)^44^ | Juvenile domestic pigs were divided into two groups:   - Normocarbia group (control group) contained 10 pigs. - Hypercarbia group (high CO_2_ group) contained 8 pigs. | - PRx: based on slow-wave changes in measured ICP taken from an invasive ventricular catheter - HVx: NIRS-based index | LLA:   - A scatterplot of 60-second averaged values of LDF versus CPP was made using the ICM+ software for each piglet. - CPP at the intersection of 2 regression lines with the lowest combined residual squared error was defined as the autoregulatory breakpoint for each animal. | Primary: Determine if hypercarbia would alter cerebral blood flow autoregulation and reduce the ability of cerebrovascular reactivity monitoring to identify the LLA. | These results might not extrapolate to adult pigs or human populations since the data was obtained in juvenile piglets.  If there are regional differences in CBF, then the measurement of CBF at a discrete location in the cortex can confound the calculation of LLA.  The study was not designed to evaluate hypocarbia effects on vascular reactivity and autoregulation monitoring and function. |
| Ruesch et al. (2021)^45^ | 12 non-human primates (NHP), Macaca mulatta, males with the age of 8.1 ± 1.7 years and weighing 9.9 ± 2.5 kg:   - 7 NHPs were isoflurane-anesthetized - 5 NHPs were fentanyl-anesthetized | PRx | CBF measured using a custom-built diffuse correlation spectroscopy system and Lassen’s curve was constructed based on the diffusion coefficient value.  Lassen’s curve calculated for each NHP before averaged in groups of isoflurane and fentanyl anesthesia. The plateau in the Lassen’s curve indicates intact cerebral autoregulation and the sloped areas below and above the plateau are LLA and ULA, respectively. | Primary: Determine how anesthetics can be used to manipulate cerebral autoregulation in NHPs and how static and dynamic autoregulation compare under challenges in ICP and MAP.  Secondary: Analyze the agreement of cerebral autoregulation measurement methods of Lassen’s curve, phase delays and PRx. | Not able to assess age and sex since the NHPs had been part of previous scientific experiments and were used in this study to reduce the number of animals in scientific experiments while maximizing their use. |
| Zeiler et al. (2018a)^20^ | Archived data of 12 New Zealand (NZ) rabbits were analyzed from previous studies. | PRx  PAx  Mx  Sx  Lx  RAC | LLA:   - Plots of LDF-CBF versus CPP or FVs versus CPP were constructed using the ICM+ software. - Breakpoint representing the LLA for each animal was identified at the intersection of 2 linear segments with the minimized sum residual squared error with the automated piecewise linear regression conducted with R statistical software. - Mean LLA was determined by averaging all 12 LLA values obtained for the cohort of 12 rabbits. | Primary: Validate PRx and ICP-derived indices (PAx, RAC) of cerebrovascular reactivity against LLA in an intracranial hypertension model. | Translation of the results to clinical monitoring in humans is limited since the response of rabbits’ cerebral vascular system is not identical to humans, but it does behave in a similar manner.  Only 12 out of 28 rabbits from original experiments were included since they had sufficient quality of archived signals for defining and evaluating the LLA.  Despite the pCO_2_ being controlled during the experiment, the standard deviation of 5.7 mmHg may still have impacted the vasoreactivity in the animal models.  No animals were studied with arterial hypotension in isolation.  There were indices that were derived from ICP and TCD (RAC, Mx, and Sx) that could not confirm or refute their association with the LLA due to failure to produce reliable data.  The rapid rise in ICP may have played a part in the noisy data, but it is unknown if the longer rise period of ICP would have provided data with less noise.  This study does not address if PRx and PAx respect the ULA but provides validating evidence for monitoring the LLA.  It is unknown if PRx maintains the ability to measure autoregulatory capacity for extreme intracranial hypertension in humans as it does in animal models.  Even though PRx and PAx appear to be related to LLA, the Bland-Altman analysis indicates poor agreement between CPP at LLA and at all index thresholds tested. |
| Zeiler et al. (2018b)^19^ | Archived data of 22 neonatal piglets were analyzed from three separate experiments:   - Control animals from a study on LLA which had 8 piglets that were 5 to 10 days and weighed 2.2 to 3.9 kg. - Sham controls for a model of cardiac arrest which had 7 piglets that were 3 to 5 days old and weighed 1 to 2.5 kg. - Sham normothermic controls for a model of cardiac arrest with hypotension therapy which had 7 piglets that were 3 to 5 days old and weighed 1 to 2.5 kg. | PRx  PAx  RAC | LLA:   - Plots of LDF-CBF versus CPP were constructed using the ICM+ software. - Breakpoint representing the LLA for each animal was identified at the intersection of 2 linear segments with the minimized sum residual squared error with the automated piecewise linear regression conducted with R statistical software. - Mean LLA was determined by averaging all 22 LLA values obtained for the cohort of 22 piglets. | Primary: Validate ICP-derived indices (PAx and RAC) of cerebrovascular reactivity against LLA during arterial hypotension. | The experimental procedures from three different experiments were similar but were not exactly identical so it may have slightly influenced cerebrovascular response.  Conclusions drawn from this study should be taken with caution since the cohort size taken was relatively small.  The results in this study mainly apply to animal models and there is limited ability to extrapolate them to human TBI care. |
| CBF, cerebral blood flow; COx, cerebral-oximetry index; COx-a, COx obtained with MAP; CPP, cerebral perfusion pressure; FVs, systolic flow velocity; HA, hypoxic-asphyxic; HVx, hemoglobin volume index; ICM+, Intensive Care Monitoring software (Cambridge Enterprise Ltd, Cambridge, UK, http:// icmplus.neurosurg.cam.ac.uk); ICP, intracranial pressure; iPRx, induced PRx; LDF, laser-doppler flow; LDF-CBF, LDF-based CBF; LDx, laser-doppler index; LLA, lower limit of autoregulation; MAP, mean arterial pressure; MAP_opt_, optimal MAP; Mx, mean flow index; NIRS, near-infrared spectroscopy; PAx, pulse amplitude index; pCO_2_, partial pressure of CO2; PEEP, positive end-expiratory pressure; PRx, pressure-reactivity index; RAC, correlation between pulse amplitude of ICP and CPP; rSO_2_, regional cerebral oximetry; Sx, systolic flow index; rTHb, relative total hemoglobin; TCD, transcranial doppler; ULA, upper limit of autoregulation; wCOx, wavelet COx; wHVx, wavelet HVx; wPRx, wavelet PRx; ΔCBF, percentage changes of diffusion coefficient (αD_b_); Δϕ_AI_, MAP-ICP phase shift. | | | | | |
